# Three-Dimensional Cell Culture Systems in Radiopharmaceutical Cancer Research

**DOI:** 10.3390/cancers12102765

**Published:** 2020-09-25

**Authors:** Alina Doctor, Verena Seifert, Martin Ullrich, Sandra Hauser, Jens Pietzsch

**Affiliations:** 1 Department of Radiopharmaceutical and Chemical Biology, Institute of Radiopharmaceutical Cancer Research, Helmholtz-Zentrum Dresden-Rossendorf, 01328 Dresden, Germany; a.doctor@hzdr.de (A.D.); v.seifert@hzdr.de (V.S.); m.ullrich@hzdr.de (M.U.); s.hauser@hzdr.de (S.H.); 2 School of Science, Faculty of Chemistry and Food Chemistry, Technische Universität Dresden, 01069 Dresden, Germany

**Keywords:** co-culture, organoids, pancreatic cancer, radiotherapeutics, radiotracer, spheroids, stromal cells, 3D model, tumor microenvironment

## Abstract

**Simple Summary:**

Cancer cells cultured in three-dimensional (3D) model systems exhibit physiologically relevant cell–cell and cell–matrix interactions, gene expression patterns, and signaling cascades as well as heterogeneity and structural complexity that more reliably reflect tumors and metastases than monolayer cultures do. In recent years, the development of various 3D models, including scaffold-free, scaffold-based, chip-based, and organoid systems, has improved, among other things, the characterization of new radioligands and the use of screening platforms for the development of radiotracers and radiotherapeutics. This review article attempts to summarize and critically discuss the suitability of different 3D culture systems in radiopharmaceutical cancer research. Specific emphasis is put on pancreatic ductal adenocarcinoma, which is a predestined target for new radiotheranostic agents. This overview also highlights the different sophisticated techniques for generating 3D models and their characteristics.

**Abstract:**

In preclinical cancer research, three-dimensional (3D) cell culture systems such as multicellular spheroids and organoids are becoming increasingly important. They provide valuable information before studies on animal models begin and, in some cases, are even suitable for reducing or replacing animal experiments. Furthermore, they recapitulate microtumors, metastases, and the tumor microenvironment much better than monolayer culture systems could. Three-dimensional models show higher structural complexity and diverse cell interactions while reflecting (patho)physiological phenomena such as oxygen and nutrient gradients in the course of their growth or development. These interactions and properties are of great importance for understanding the pathophysiological importance of stromal cells and the extracellular matrix for tumor progression, treatment response, or resistance mechanisms of solid tumors. Special emphasis is placed on co-cultivation with tumor-associated cells, which further increases the predictive value of 3D models, e.g., for drug development. The aim of this overview is to shed light on selected 3D models and their advantages and disadvantages, especially from the radiopharmacist’s point of view with focus on the suitability of 3D models for the radiopharmacological characterization of novel radiotracers and radiotherapeutics. Special attention is paid to pancreatic ductal adenocarcinoma (PDAC) as a predestined target for the development of new radionuclide-based theranostics.

## 1. Introduction

**Figure 1:**
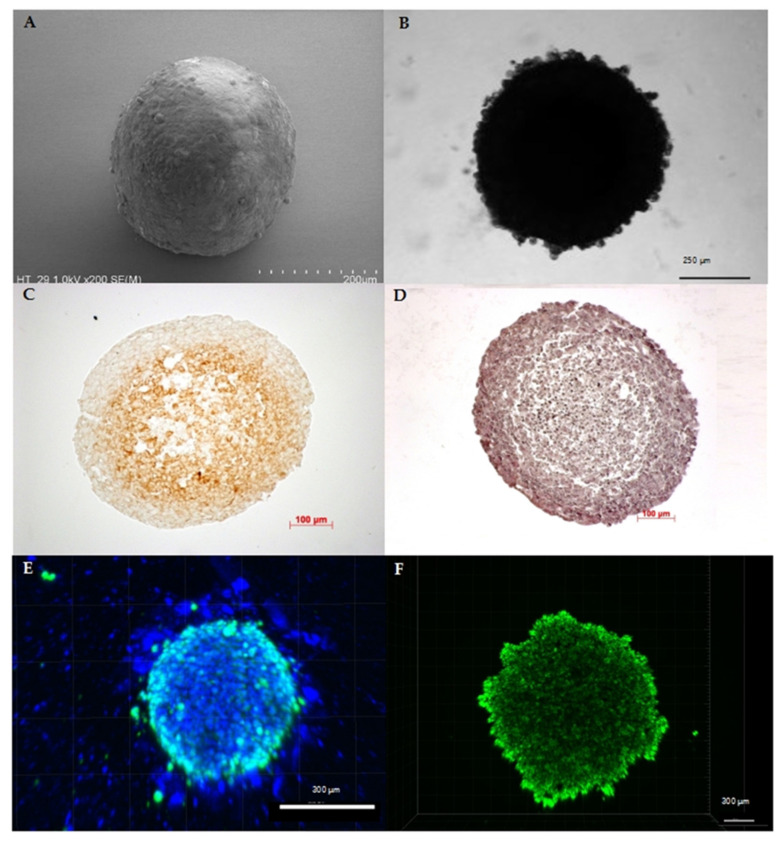
Characteristics of spheroids originating from human cancer cell lines: (**A**) SEM image of a colorectal cancer cell line (HT29) spheroid depicting the spherical shape as the main characteristic of spheroids. (**B**) Bright-field microscopy image of a pancreatic cancer cell line (HPAF II) spheroid confirming stability of the 3D cell assembly. (**C**) Like tumors in vivo, spheroids show physiological zoning, consisting of a rim of viable cells and, depending on their size, of hypoxic or necrotic core zone. Exemplarily, the hypoxic core of a melanoma cell line (A2508) spheroid with a diameter of approximately 0.55 mm is shown by pimonidazole staining. (**D**) Hematoxylin and eosin staining of a melanoma cell line (A2508) spheroid showing the heterogeneous composition that can vary in cell and extracellular matrix arrangement depending on cell line and treatment. (**E**) Distribution of viable cells and, as a further characteristic feature, the possible active migration of cells from the 3D cell assembly: here, exemplarily shown by fluorescence staining with DAPI (4′,6-diamidino-2-phenylindole) (blue) and calcein (green) in a 10-day-old co-cultivated spheroid of pancreatic carcinoma cell line (SW1990) and pancreatic stellate cells (HPaSteC) with an initial seeded cell number of 16,000 cells (ratio 1:3). (**F**) Spheroids are characterized by the formation of gradients, which, among other things, modify the transport of nutrients, metabolites, and drugs similar to the situation in vivo, as shown here in the passive transport of the fluorescent marker calcein in a 10-day-old pancreatic cancer cell line (PanC-1) spheroid with an initial seeded cell number of 16,000 cells.

For 50 years now, since their introduction in 1970 by Sutherland et al. [1], so-called spheroids of various cancer cell lines have found their place in cancer research as three-dimensional (3D) tumor models. Today, such 3D models are basically separated into three main categories: scaffold or matrix-based, scaffold-free, and organoids. Multicellular tumor spheroid (MCTS) as a term is unfortunately not consistently defined throughout the literature [2]. Sometimes loose packages of cells that aggregate but do not form compact structures are misleadingly described as spheroids [1,3]. Therefore, we like to emphasize that, in this review, the term spheroid is used exclusively for tight cell aggregates that possess a spherical shape, are stable, and can be lifted or relocated without disintegration (Figure 1).

Moreover, we will describe the possibilities and advantages of 3D models, especially with regard to the ductal pancreatic adenocarcinoma (PDAC). Since there is no characteristic symptom, PDAC is a very deceptive and ultimately very dangerous cancer. PDAC is the deadliest tumor entity worldwide with an overall 5-year survival rate of only about 7%. The mean survival time in PDAC is 6 months after diagnosis, which is extremely short. In almost all cases, it forms metastases, primarily in liver and lungs, and only a small percentage of patients (15–20%) benefit from resection [4,5,6]. The PDAC etiology is highly complex, as little is known about the risk factors and the characteristics of precursor lesions. PDAC is a very heterogeneous tumor-stroma entity with up to 90% of the tumor mass formed by stromal cells [5,7]. The tumor stroma is mainly created by activated pancreatic stellate cells (PSC) [8]. In the healthy pancreas, nonactivated PSCs are located around the acinar cells, store lipid droplets containing vitamin A, and synthesize proteins of the extracellular matrix (ECM). Upon activation, PSCs not only lose their vitamin A storing function but also start producing extensive quantities of ECM proteins leading to fibrosis. Various factors lead to PSC activation such as alcohol; oxidative stress; overexpression of various factors, e.g., cyclooxygenase-2 and fibrinogen; as well as hypoxia [9]. The large proportion of stromal cells leads to pronounced resistance to irradiation and chemotherapy of these tumors [4,10]. A major challenge is the development of early diagnosis imaging tests and more effective therapeutic approaches. Hence, for a fast and successful establishment of new radiotracers, drugs, and potentially also radiotherapeutics, it is essential to use models that reflect the tumor’s distinctive features already at the in vitro cell culture level. Various spheroid- and organoid-based approaches have proven to be particularly suitable for modelling PDAC in vitro, which will be discussed in more detail below.

## 2. In Vitro 3D Models Are Superior to Classical Monolayer Cultures

In the following, three-dimensional cell culture models in vitro are referred to as “3D models”. Moreover, the term “spheroid” is used synonymously for MCTS. Three-dimensional models are well known to overcome at least some of the limitations of classical monolayer cultures. For example, 3D models mimic biological situations better than classical cell cultures, as monolayer cultures adapt extensively during 2D culture in vitro. Rare clones expand and proliferate, causing the cell line to undergo substantial genetic changes that no longer represent the genetic heterogeneity of human tumor [11].

Through genomic profiling of parental tumor, monolayer, and spheroid cultures of human glioblastoma, De Witt Hamer et al. concluded that spheroids are more representative of the parental tumor. This hypothesis is based on the average correlation coefficient of the genomic profiles between the culture method and the original tumor, which was higher for spheroids (0.89) compared to monolayer cultures (0.62). In addition, half of the analyzed monolayer cultures showed substantial genetic changes even in short-term culture [12]. Spheroid cultures, especially from primary tumor cells are therefore genetically more stable [3,12,13].

Besides genetic variance, monolayer cultures differ morphologically [14,15] and metabolically [16,17]. While 2D cultured cells possess an unnatural stretched form [14,15]. Three-dimensional cultured cells keep the same distinct morphology [15] and cell density [18] as natural tissue. Along with morphology of single cells, the spatial cell organization of the whole cell aggregate plays a certain role in mirroring the 3D context of relevant physiological gradients of tumors in vivo due to nonuniform oxygen and nutrient levels [3,19].

Among the processes that can be better visualized and studied with 3D models are phenomena associated with hypoxia. Near the surrounding media, the outer layer of a spheroid consists of proliferating cells while the spheroid core hosts quiescent, hypoxic, and necrotic cells [1,3,14,20,21]. These innermost cells die via apoptosis due to limited availability of oxygen, nutrients, and growth factors [1,14,21]. Similar to tumor xenografts, large spheroids of about 400 to 500 µm [22,23,24,25] comprise up to 20% hypoxic cells [26]. Hypoxia in solid tumors occurs at a distance of approximately 75–80 µm [27,28] from functional blood vessels, and it is generally accepted that tumors do not exceed 1–2 mm in diameter without the induction of angiogenesis [27]. Similar to solid tumors, the complex tissue-like structure derives from the 3D physiological organization that is dependent on cell-matrix interactions and variations in the supply and transport of glucose and lactate as well as gradients in pH and oxygen [22,29]. Pronounced gradients form in particular in PDAC as an extensive ECM creates barriers that physically inhibit penetration [30,31] and cause interstitial pressure resulting in vascular compression and decreasing molecular transport [31,32]. Monolayers though, lose tissue-specific architecture that is responsible for the biochemical gradients and cell-cell and cell-matrix interactions [21]. No gradient can form in monolayer culture, as all cells receive a homogenous amount of nutrients and growth factors through the surrounding media. Moreover, the culture just consists of proliferating cells as necrotic cells are removed by regular media changes [14].

The development of secondary central necrosis was studied in more detail over the past decades, showing that apoptotic and necrotic pathways are highly relevant. According to Mueller-Klieser [25], spheroids can be classified into three groups that differ in timing of hypoxia and necrosis. Either hypoxia precedes the appearance of necrosis, necrosis precedes hypoxia, or they emerge simultaneously. As spheroids express micro-regions with different cell phenotypes depending on spatial distance from nourishing capillaries, they resemble poorly vascularized, hypoxic solid tumors with their cellular environment and behavior more closely than monolayers [13,14,21,29]. The cell viability in spheroids is preserved despite the gradients. In the beginning of spheroid formation, cells proliferate, macromolecules are synthesized, and genes are active or are activated. Those processes have a high demand for ATP with the result that a high metabolic rate must be maintained despite oxygen and substrate limitation processes. While spheroids grow, a necrotic core begins to form, which induces a decrease in the oxidative phosphorylation rate. To counter the decrease, the glycolytic uptake increases fourfold. While in small spheroids with a size of 0.4 to 0.5 mm, oxidative phosphorylation generates the majority of ATP; in larger spheroids with a size of 1 mm, glycolysis produces most of the ATP. Although ATP production is lower in larger spheroids, it is enough for survival [29]. The change in the ATP-generating pathway in spheroids is accompanied by a more than threefold increase in hypoxia-inducible factor 1-alpha (HIF-1α) expression [29]. The reduced proliferation rate in older spheroids, in contrast to always proliferating monolayer cultures, is more similar to the situation in vivo [14]. The 3D environment with its complex cell-cell and cell-matrix interactions affects not only gradients but also cell surface molecules leading to altered intracellular signaling pathways [33].

The described metabolic and proliferative gradients in vivo contribute to the therapeutic challenges of tumors [3]. Because of its similarity to tumors, spheroids are the ideal link in complexity between monolayer culture and in vivo models [26]. All these advantages are also beneficial for theranostic research for PDAC, allowing for investigations not only on altered gene expression and protein levels but also on responses to treatment.

## 3. Current Methods and Challenges in Cultivation of Multicellular Spheroids

There are two principally distinguishable methods to produce spheroids: scaffold-free and scaffold or matrix-based (Table 1). The overall aim is to efficiently and conveniently produce uniformly sized 3D tumor models that are suitable for the desired application such as detection of protein levels or drug screening [13].

Scaffold-free methods comprise approaches from forced-floating to agitation-based methods as well as the hanging drop method. The term “forced-floating” involves approaches that use low-adhesion plates. Cell culture plates are coated with 0.5% poly(2-hydroxyethyl methacrylate) (PHEMA) [1,13,33,60], or 1.5–3% agarose [1,33] to prevent cells from attaching. Cell–cell interactions develop, and as a consequence, spheroids are formed [33]. This method is straightforward and produces consistent spheroids with diameter variation below 5% [1,33]. Spheroid size is adjustable depending on the cell number in each well. Cultivation of spheroids in multiwell-plates allows high-throughput screening and easy accessibility [21,33].

Agitation-based methods retain the cells in constant motion so that the cells do not attach to the walls of the container but interact with adjacent cells [20]. Alternatively, preestablished spheroids produced in low-adhesion plates can be used [1]. Long-term culture of these spheroids is possible, as media change is simple. Constant motion also allows easy transport of nutrients and waste [20]. On the other hand, motion generates sheer forces potentially affecting spheroid morphology and size. Consequently, agitation-based approaches show relatively high variation in spheroid size [33] and might therefore be inappropriate for drug testing. Besides, the large media volume is unfavorable for drug screening, as only minimal amounts of drug candidates are often available [1].

Another important approach is the hanging drop method. A drop of cell suspension is placed onto the underside of a culture dish lid. Spheroids form, as gravity accumulates the cells at the tip of the drop [13,21,33]. With this method, spheroids are simple to establish and reproduce; however, the drop is limited to a small volume and media change is difficult [33]. Therefore, some advanced versions, for example by Tung et al. [61] or Hsiao et al. [45], use specialized plates. Tung et al. created a 384-well plate with holes in the top lid allowing direct access and therefore long-term culture and high-throughput drug screening [61].

The overall advantage of scaffolds is that they provide a physical structure comparable with the extracellular matrix in vivo [34,62]. Scaffolds should be porous to allow oxygen, nutrient, and drug transport as well as waste removal [33,63]. The most common scaffolds are Matrigel™, hyaluronic acid, polyethylene glycol (PEG), and polyvinyl alcohol (PVA) [14,21]. Besides these, fibrin, chitosan, alginate, and silk fibrils can be used but are less versatile [21]. Biological matrices promote cell organization as they contain hormones and soluble growth factors. However, composition may vary between different batches [21,33] and the additional growth factors may alter experimental results. Matrigel™ for example consists of extracellular matrix obtained from Engelbreth–Holm–Swarm mouse tumor sarcoma. The matrix contains basement membrane proteins such as collagen IV, laminin, perlecan, entactin, matrix metalloproteinase-2 (MMP2), and growth factors. For pancreatic cells, the most widely used materials are collagen and Matrigel™ [34,35,36]. To mimic pancreatic tissue, a combination of 75% collagen I and 25% Matrigel™ can be used [37]. Hyaluronic acid is an example of natural scaffolds without eukaryotic growth factors, as it is commercially produced by bacteria [62]. Synthetic scaffolds maximize reproducibility and provide possibilities for tuning biochemical and mechanical properties [21]. However, they are not as biologically relevant as natural scaffolds [62]. Furthermore, scaffold materials based on hydrogels are high in water content and enable transport of soluble factors [21]. Methylcellulose thickens cell suspensions and is therefore used for cells that do not form spheroids in scaffold-free methods as it is the case for many pancreatic cancer cell lines [38]. Consequently, methylcellulose counts as scaffold even though, in some research articles [64], 3D cultivation with methylcellulose is misleadingly described as a scaffold-free method.

Spheroids have been used in different fields of preclinical research to investigate, amongst others, regulation of gene expression and protein levels [40,65,66] (Table 2).

## 4. Organoids as a Step Forward to Personalized Medicine

Besides multicellular spheroids, organoids are praised as an efficient tool for cancer research. In contrast to spheroids originating from previously established cell lines, organoids grow from cells obtained directly from healthy or tumorous tissue or stem cells [11,21,34,76]. Organoids retain functionality and appearance of the original tissue [76].

Similar to other 3D culturing methods, organoids are grown encapsulated or on top of scaffolds such as collagen or Matrigel™ [34,76]. Together, scaffold material and media containing growth factors mimic a natural, growth promoting cell environment [77]. In this manner, human pancreas duct cells can be cultured in vitro up to five months [46] while remaining genetically stable [78]. Organoids derived from healthy pancreas progenitor cells show normal pancreatic development with differentiation into exocrine and endocrine cells [79]. Prior to seeding, tissue must be dissociated either enzymatically or mechanically [34,46,76]. Methods for creating pancreatic organoids are for example described by Broutier et al. [46], Huch et al. [47], and Boj et al. [48]. Besides establishing organoids from cancerous tissue, oncogenic mutations of interest can be introduced into cells [49,80]. In the case of PDAC, the *KRAS* mutation is the most common mutation at 90%, is responsible for stimulated proliferation of cancer cells, and occurs early in tumor formation [80]. Therefore, introducing a *KRAS* mutation in cells is a step towards understanding its role in PDAC progression [69]. Further applications for organoids as well as spheroids in PDAC research can be found in Table 2.

Organoids are suitable for biobanking. For that purpose, large amounts of organoids derived from cancerous tissue are grown and stored for further research [11,76]. Some of these investigations focused on potential relationships between genetic variation and drug efficacy. Here, biobanked, cryopreserved organoids ensured access to statistically relevant sample numbers.

Moreover, organoids are relevant for personalized medicine. Treatment responses of healthy and tumorous organoids are evaluated to choose the best possible therapy for a patient by considering the patient’s genetic background. Ideally, treatment should eradicate the tumor cells while leaving healthy cells undamaged. To test this, two organoids per patient are established. One organoid originates from healthy tissue, and the other originates from tumorous tissue. Based on proteomic data obtained from both organoids, tumor-specific characteristics across all patients and patient-specific characteristics can be analyzed. Changes in protein levels can further be assigned to changes in cell signaling pathways [81].

Apart from individual treatment planning, also effects of novel drugs can be assessed [82]. Some small-scale drug screenings revealed encouraging results [11]. To establish an organoid culture, just a small number of cells is necessary. Therefore, cells can be gained not only from resected tumors but also by endoscopic fine-needle aspiration [46,48,76,78]. This is of particular interest for research on PDAC, with just 15–20% of patients undergoing a resection of the tumor [5,6,83]. First results on drug screens and assessment of biomarkers are available three to four weeks after surgery [76]. During this reasonably short time, the risk of genetic changes within the organoids is relatively low. Therefore, use of organoids ensures that treatment evaluation is performed in models maintaining as much as possible the characteristics of the primary cancerous tissue [84].

## 5. Co-Cultures Visualize Challenges Associated with Tumor–Stroma Interactions

In pancreatic cancer, up to 90% of the tumor mass is formed by stroma, which is created by activated pancreatic stellate cells (PSC) and is responsible for the unique microenvironment of pancreatic cancers [85,86]. There are multiple experimental and clinical evidences that the microenvironment of pancreatic cancers is mostly responsible for resistance to chemotherapeutic agents [87,88], targeted drugs [89], immunotherapy [90], and radiotherapy [91]—a characteristic that is also referred to as environment-mediated resistance [92].

To visualize the effects of tumor–stroma interactions, particularly with regard to therapeutic resistances of PDAC, 3D models need to reflect the complex and heterogeneous composition of the ECM. As a key component of tumor stroma, the ECM strongly influences the behavior of tumor and stroma cells in fibrotic tumors such as pancreatic cancer [31,37]. In our opinion, this effect should not be artificially altered by adding additional matrix components such as Matrigel™, as it has been shown that matrix components influence the epithelial–mesenchymal transition (EMT) of PDAC cell lines [93]. Therefore, a model is needed that includes the effects associated with the stroma, however, without altering the situation with external factors. Until now, the models most suitable for meeting this challenge are co-culture 3D models, as they include other cell types besides cancer cells.

Co-culture models are used to test the effects of neighboring non-tumor cells on 3D systems [62]. As tissues comprise more than one specific cell type, 3D models that include the heterotypic composition of tissue are most likely mimicking the in-vivo situation [37,50,94]. Depending on the author and source, those models are sometimes called co-culture spheroids [95], heterotypic spheroids [13], organotypic cultures [37], tumoroids [96], hybrid spheroids [97,98], or heterospheroids [72]. In this review, the term co-culture spheroid is used. Despite the differences in term, they all have in common that (cancer) cells are cultured together with other cells, e.g., immune cells [3] or stromal cells such as fibroblasts [1,50,72]. Co-culturing with immune cells such as macrophages, monocytes, etc. is used for studying migration and activation in the field of immunotherapy [3], while the stromal environment is interesting for inquiring effects on drug administration and radiotherapy [1,62,97] as well as tumor cell invasion and metastasis [13,62]. The interaction of tumor cells and associated cells is essential for mimicking tissue architecture in vivo [13,37,62] including vascularity [99,100].

Co-culturing pancreatic cancer cells together with stromal cells increased the invasiveness of tumor cells by 4- to 16-fold [50,101] and boosted therapy resistance [68,72]. These changes are most likely due to signaling events between the two cell types [101].

Even in 2D cultures, similar results were obtained by co-culture of pancreatic cancer cells with pancreatic stellate cells, where an increased migration and altered expression of EMT marker genes were observed [74]. These observations are even more pronounced in 3D co-cultures [51]. Taken all together, the effects of co-culturing pancreatic cancer cells with other cells increased proliferation [73], decreased T cell proliferation [52], and changed gene expression and protein levels with effects on signaling pathways [37,73]. Furthermore, the co-culture spheroids showed environment-mediated resistances [54,73] and increased invasiveness [50] (Table 2). Another aspect of co-culturing to take into account is the spatial organization of the cells in a spheroid. Wong et al. as well as Firuzi et al. observed that, in the co-culture of pancreatic cancer cells and PSC, the PSC forms the outer layer of the spheroid while the cancer cells form the core [41,68]. This contradicts the findings of Norberg et al. describing the reverse case [64]. Further research is needed to verify which result truly reflects the situation in vivo.

For several cancer entities, the co-culture method can be enhanced by cultivating three cell types in one model [102,103]. The benefits of so-called triple-cultures apply for PDAC as well. The effect of pancreatic cancer cells and fibroblasts on a third cell type, e.g., monocytes or endothelial cells, can be evaluated [52,54], aiming to reflect cancer progression in patients [50,52].

There are multiple approaches for setting up a co-culture experiment. So-called mixed spheroids are established by mixing tumor and other cells, e.g., fibroblasts in the desired ratios prior to spheroid formation in plates [1,3]. Another approach is to place already formed tumor spheroids on top of a monolayer of associated cells [1,3]. A third method is to put tumor spheroids and spheroids of associated cells side by side to allow for overgrowth [104,105]. By placing a spheroid of associated cells in a suspension of tumor cells, the migration of not yet aggregated tumor cells into adjacent tissue can be observed [1,106].

In summary, co- or even triple-cultured spheroids exhibit strong similarities with tumor tissue in patients, enhancing further the already existing advantages of spheroids compared to monolayer culture for drug development.

## 6. Biotechnical Microsystems Enable New Design of 3D Models

**Figure 2:**
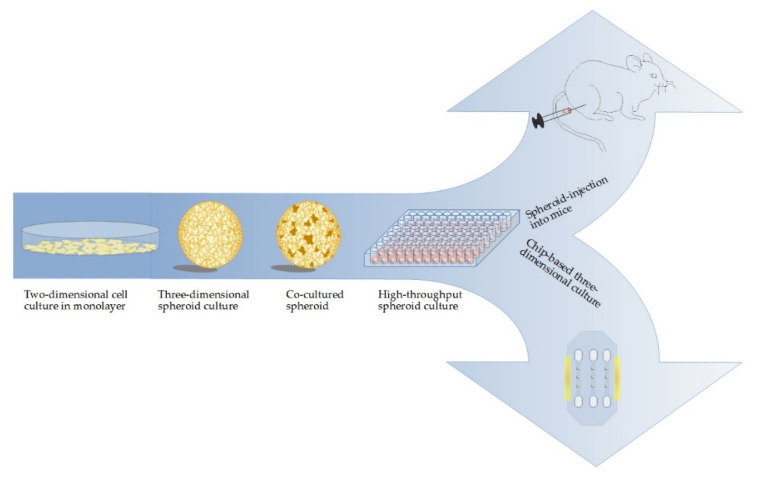
Increment of complexity in cell culture methods for PDAC research: cancer research is moving forward from monolayer cultures towards the use of spheroids. Including a second cell line, e.g., fibroblasts, into the model further increases the complexity. One-step further, spheroids are injected into mice for an advanced model in vivo with the possibility to mimic the progression of small metastases to tumors. On the other hand, using biotechnological state-of-the-art, for example HepaChip^®^, allows for measurement of cell reactions directly with electrodes.

Besides these “classical” approaches of 3D cell-culture, new approaches have been established including “Tumors on a strip” [75], cell-based biosensors [14], and chip-based approaches [13,14,21,107,108] (Figure 2). These device-based 3D models bear some advantages over spheroid and organoids, including reproducibility and real-time measurements directly inside the cultivation device [109].

For instance, in 2015, Rodenhizer et al. [75] presented the “Tumor Roll for Analysis of Cellular Environment and Response” (TRACER), a device-based 3D model where collagen-embedded tumor cells grow on a cellulose strip. This strip is rolled onto a core and dipped into cell culture medium. Unrolling of the strip then allows for collection of cells from the desired parts of the tumor model and rapid analysis. As in the other 3D cultures, the tumor-on-a-strip develops nutrient and oxygen gradients and is therefore suitable for analysis related to hypoxia.

Two-dimension-based microelectrode arrays, well-known in neuronal network studies [110,111], can be applied for 3D models, e.g., for chips [112] or cell-based biosensors [113]. These approaches require attached, embedded [112], or trapped [114] cells on the substrate. For directly attaching the cells to the substrate, extracellular matrix proteins such as fibronectin or collagen can be used [112]. Popular embedding materials are hydrogels such as PEG [112,115] or alginate [116]. Trapping can be implemented by fixing the spheroid between two electrodes. The density of spheroids trapped between two gold electrodes can be measured, as high-density correlates with low amplitudes at high frequencies. Therefore, the effect of drug candidates on the spheroid density can be evaluated [114]. The surface material is usually silicon, glass, or plastic. The advantage of transparent materials is that investigations using, for instance, fluorescent microscopy are possible [112].

Another method is the cultivation of spheroids on top of small pillars. The plastic pillar is first coated with Matrigel™; then coated with poly-L-lysine and BaCl_2_ (for crosslinking); and then dipped into a cell suspension with 1% alginate [117], collagen, or Matrigel™ [118]. Similar to the hanging-drop method, spheroids form on the tip of the pillar by inverting and dipping it into media. This method allows easy media change and treatment testing by dipping into drug containing media. As the pillar inserts are compatible with 96-well plates, high-throughput analysis is possible [117]. The spheroids can be used afterwards for histology by embedding them in paraffin or by freezing in liquid nitrogen [118].

Due to their sensitivity to changes and the possibility for high-throughput drug screening in vitro, whole cell-based biosensors are applied in cancer research (Table 2) [119]. Some research groups also combined the ideas of co-cultivating spheroids with device-based methods [55,56]. It should be mentioned that, in those approaches, the cells are not truly cultured together as in spheroid co-cultures but cultivated together on a microfluidic chip with close proximity to each other, divided by a small channel between them, filled with media. Through the media, the two co-cultured cell types are allowed to communicate and migrate [55]. Because the cells do not have direct contact, the setup is less a co-culture model than a model with conditioned media. This setup is used for example to culture pancreatic cancer cell spheroids together with stellate cells in collagen-coated microchannels on a plate [56]. The microfluidic chip used in this setup is called HepaChip^®^, consisting of a cyclic olefin polymer chamber coated with collagen. PDAC cells cultivated on those chips have been reported to be vital and to appear morphologically similar to cells in spheroids [57,58]. With the help of this chip, response to treatment with cisplatin has been monitored [57]. The distance between cell types can be adjusted by geometry and arrangement of the microchannels [95]. Under these experimental conditions, changes associated with co-culturing such as migration activity, drug resistance, EMT marker expression [55,56], morphological changes, and increased spheroid size [55] were observed. The microchannels represent vascularity in a tissue and therefore integrate a stable nutrient supply and shear stress into the model [120,121].

A further method is the magnetic 3D bioprinting of cells into microplates. Biocompatible nanoparticles are attached to plasma membranes of cells. The magnetized cells are then printed into multi-well microplates. The nanoparticles are released within one week. This strategy was applied to pancreatic cancer cells and activated pancreatic fibroblasts, producing easy and reproducible spheroids that can be used for drug screening [59].

## 7. Translation of 3D Cell Culture Models from In Vitro to In Vivo

Besides establishing 3D models in vitro, it might be interesting for some applications to transplant preformed 3D structures into animals. Both spheroids and organoids may serve this purpose. Transplanted organoids have been shown to retain features of the original tumor [11]. For pancreatic organoids, successful transplantations either orthotopically or subcutaneously have been reported. For orthotopic transplantation into the pancreas, the 3D structure needs to be dissociated. Then, either small cell aggregates of approximately 1 × 10^5^ cells [70] or a cell suspension [71] are injected. Orthotopically transplanted organoids in mice have been reported to create preinvasive lesions that resemble pancreatic intraepithelial neoplasia (PanIN) [48,76], discussed as a precursor of PDAC [122]. Additionally, after forming PanIN-similar lesions, the organoids progress into invasive PDAC [48]. Therefore, orthotopic transplantation into the pancreas might be a model for the early stage of tumor formation (Table 2).

However, a major advantage of subcutaneous compared to orthotopic transplantation is that organoids or spheroids do not need to be dissociated. A small incision into the skin allows for transplanting the 3D structure into the space between skin and muscle and then for suturing the wound [70]. With this technique, smaller 3D aggregates can also be injected. Subcutaneous xenograft models based on injection of single-cell type spheroids and co-culture spheroids consisting of pancreatic cancer cells and fibroblasts have been reported to develop reproducible tumors which resemble natural PDAC [48,85,123]. Xenograft models of other tumor entities derived from 3D structures also resembled the clinical presentation [123,124]. Besides successful tumor formation via transplantation, also healthy pancreatic ductal tissue can be formed by transplantation of healthy pancreatic cells [48].

## 8. 3D Models as Pharmacists’ Tool for Drug Development

Three-dimensional models are acknowledged as a potential bridge between monolayer cultures in vitro and animal testing in vivo [3,33,62]. That is because 2D models generate misleading results with limited predictive value for clinical efficacy. Monolayers often fail to simulate the biological situation in tumors, and therefore, many drugs fail in clinical trials [1,11,14,29]. Almost all anticancer drugs are less effective in a multicellular spheroid model compared to monolayer cultures [125,126].

**Figure 3:**
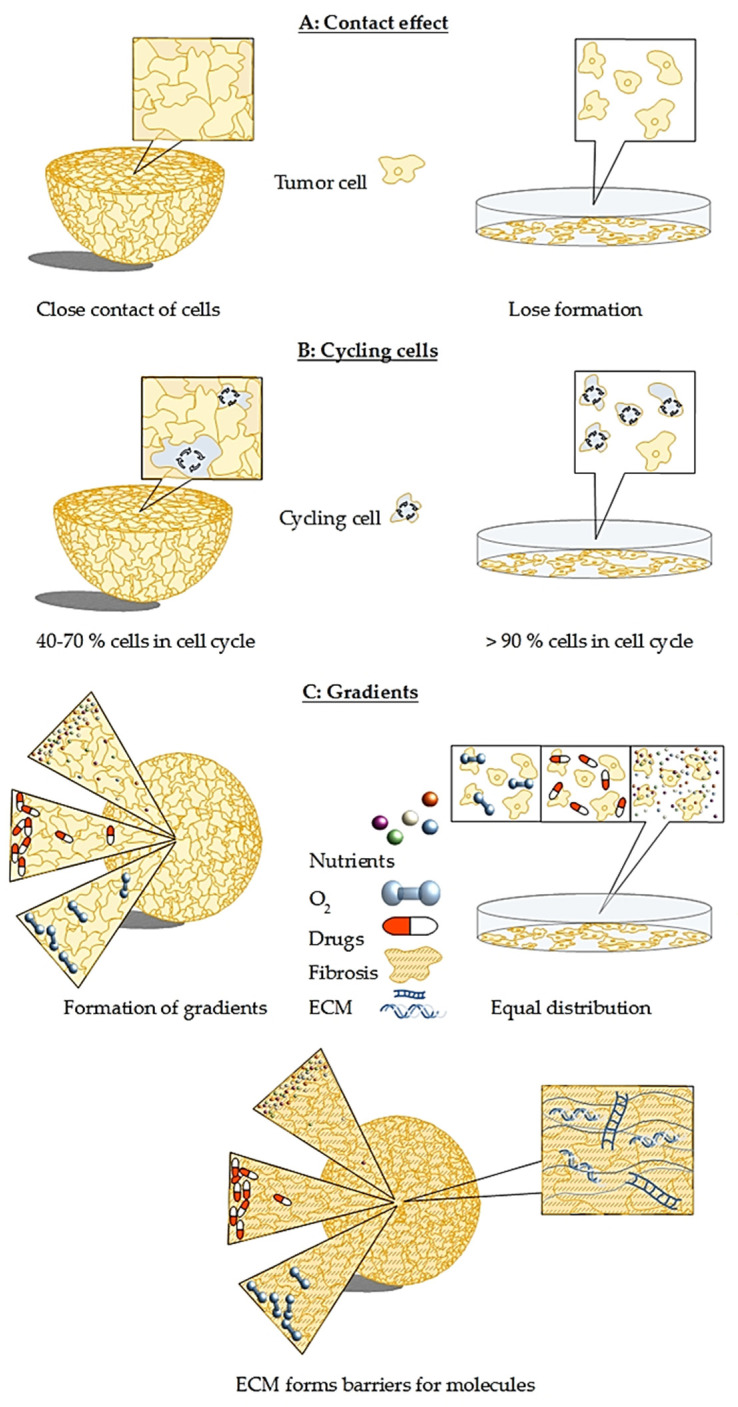
Major differences between spheroid and monolayer culture that cause challenges for therapy development: (**A**) in contrast to monolayer culture, cells in a spheroid are tightly packed. This leads to the contact effect that induces radioresistance observed in 3D models as well as in patients. (**B**) The percentage of cycling cells in spheroids is size-dependent and varies between 40 to 70%, while in monolayer culture, more than 90% of cells are cycling. That makes the monolayer cells much more prone to medication and leads to misleading results, explaining the high number of failed clinical trials. (**C**) Oxygen, nutrient, and drug gradients are present in spheroids with concentration and penetration of all three decreasing towards the spheroid core, whereas in a monolayer culture, oxygen, nutrients, and drugs can reach each cell with equal efficiency. This leads again to false expectations on the true effectiveness of a drug candidate. In PDAC, the situation is even more severe, as fibrosis leads to a dense mesh-like network that hampers nutrients, drugs, as well as oxygen from entering the spheroid, leading to pronounced gradient formation. All three challenges are present and even more complex in co-culture spheroids.

Altered treatment response of spheroids compared to 2D culture is considered a combination of three major changes: first, the higher resistance in combination with reduced drug diffusion; second, the influence of the altered cellular environment [33]; and third, the limited percentage of proliferating cells which are preferentially targeted by cytotoxic drugs [125,126] (Figure 3). In term of cytotoxicity and treatment efficacy, the predictive value of spheroid assays is therefore higher compared to monolayer cultures [17,21,126]. Additionally, 3D co-cultures of various cancer cells including pancreatic cancer cells are even more resistant to a variety of compounds than 3D cultures consisting of just one type of cells [68,72,73]. For instance, standard drugs for the therapy of PDAC such as gemcitabine and oxaliplatin need to be applied at 200-fold higher concentrations to reach the same IC_50_ value in spheroids compared to monolayer cultures [68]. Due to that phenomenon, it has been advised to test the effectiveness of treatments not only on 3D models but also on 3D co-culture models to identify compounds that are effective in both experimental setups [68]. This minimizes the costs of treatment testing, as ineffective compounds are dismissed already before animal testing.

Besides so-called negative selection, referring to the dismissal of ineffective compounds, also positive selection of substances occurs. Some targets or pathways are enhanced in the 3D environment and are therefore good targets for treatment [3,14]. An example of positive selection is the phosphatidylinositol 3-kinase (PI3K) inhibitor wortmannin and the wortmannin analogue PX-866, which was ineffective in monolayer culture but suppressed spheroid growth of, e.g., glioblastoma, prostate, breast, and colon cancer cell lines. Importantly, inhibition of spheroid growth correlated with results in human tumor xenografts [127]. Likewise, the proteasome inhibitor PS-341 showed equal or higher inhibitor potential in spheroid ovarian and prostate cultures [126]. The list of drugs with positive selection can be continued and has been reported elsewhere [61,125].

As already mentioned, depending on their size, spheroids often exhibit a hypoxic core. This is an important feature for investigations on hypoxia-selective cytotoxic compounds such as tirapazamine derivatives [10]. Besides the presence or absence of the hypoxic core, also the size of the spheroid impacts treatment testing, as some factors are spheroid-size-dependently expressed [67]. Before the start of an investigation, it is therefore of great importance to predefine besides the required statistically relevant sample numbers also the spheroid size [128,129].

## 9. 3D Models as Radiopharmacists’ Tool for Development of Radiotheranostics

Recent radiopharmaceutical research increasingly uses 3D models. The main objective is the in vitro characterization of novel radiotracers for nuclear medical imaging using positron emission tomography (PET) or single photon emission computed tomography (SPECT). Secondly, the development of targeted radiolabeled molecules with suitable radionuclides, in this case β^−^-or α-emitters, results in radiotherapeutic agents for endoradionuclide therapy for which treatment effects can be tested in 3D models. Thirdly, in order to improve radionuclide-based therapeutic approaches, neoadjuvant therapy with radiosensitizers or, with regard to the risks of normal tissue damage, radioprotectants are increasingly discussed [130]. In this regard, well-designed experiments with these models contribute to the 3R principles of animal research (replacement, reduction, and refinement).

However, the insensitivity of 3D models and tumors towards ionizing radiation remains challenging. Radiosensitivity depends on cell contact, signaling pathways, and apoptosis [10]. These factors are influenced by the three-dimensional structure of the tumor or 3D model. Moreover, hypoxia in spheroids and solid tumors results in up to 3-fold enhanced radioresistance [10,19,125,126].

For successful irradiation treatment, principles also known as the four Rs of radiotherapy must be considered: reoxygenation, redistribution, regrowth or repopulation, and repair. During irradiation, some cells die immediately, which leads to improved availability of nutrients and oxygen for the remaining viable cells. Reactive oxygen species generated during irradiation treatment are able to damage previously undamaged regions, leading to reoxygenation of surviving cells [131]. The term redistribution refers to the reorganization of cells in different cell ages as a result of selective killing of quiescent cells during therapy [26,131], causing resynchronization of the cell cycle. Due to the phenomena of reoxygenation and reorganization, radiotherapy in clinical routine is divided into several fractioned doses [26]. After therapy, only healthy cells should regrow to repair the damaged tissue. However, there is often also regrowth of tumorous cells. Similar effects can be observed in spheroids after radiation treatment [131,132].

Furthermore, phenomena such as the “contact effect” also known as “bystander effect” [58,91,92,133,134] drives radioresistance in tumors and spheroids (Figure 3). One proposed mechanism is rooted in better intercellular communication, changed cell and nucleus shape, tighter DNA packaging, and altered gene expression [135,136]. Zschenker et al. [137] suggested that increased radioresistance is likely caused by increased expression of genes for tissue development, adhesion processes, and cell defense as the authors did not observe increased expression of DNA repair genes.

Cell cycling in tumors corresponds with uptake of radiopharmaceuticals and is therefore the major limitation for targeted radiopharmaceutical therapy. In monolayer cultures, more than 90% of cells are cycling, while in spheroids, the amount of cycling cells decreases with increasing size. The fraction of cycling cells decreases size-dependently from 70 to 40% (Figure 3) [138]. It was shown that slow growing spheroids have less cells in the radiosensitive G_2_-M phase [139] and are therefore more resistant towards radiation-induced therapeutic effects [140].

Tumor spheroids are suitable for testing response to irradiation as they share similarities with microregions in a larger tumor [141]. Moreover, they show responses to irradiation similar to tumors in vivo and are therefore suitable models for irradiation experiments [10]. Interestingly, cells of a spheroid separated before irradiation treatment still exhibit radiation resistance [135]. Spheroids allow for remodeling of the cell cycle synchronization as also observed in tumors responding to irradiation or antitumor compounds. Cell cycle synchronization results in a higher radiosensitivity and is consequently associated with increased treatment efficacy (Figure 3). Therefore, spheroids can be used to predict appropriate time points with maximal sensitivity to irradiation [141]. Moreover, dose-responses of spheroids correspond to those observed in xenograft studies [26]. Furthermore, spheroids allow for testing of radiosensitizing agents. Radiosensitizers can boost the therapeutic effect of radiotherapy, e.g., by sensitizing the cancer cells to radiation while sparing healthy tissue [142].

The aspects considered here are directly related to radiopharmaceutical therapeutic developments. The main restriction of radiotherapy is the limited radiation tolerance of surrounding tissues. Targeted endoradiotherapy can limit the damage to healthy tissue. With the help of radiolabeled drugs, radiation dose is specifically targeted to and deposited in tumor cells while damages to adjacent normal tissue are minimized (Table 3). To simulate penetration, required dose, distribution, and specific concentration in tumors, spheroids are considered the ideal model in vitro [143]. For targeted radiotherapy, retention time, penetration, and physical properties of nuclides are of importance, as the absorbed energy depends on the mean path length of emitted particles [144,145]. Diagnostically and therapeutically useful isotopes including half-life times and main types of decay have been summarized in a review by Sihver et al. [146]. Alpha particles deposit their energy in a short range of up to 100 µm with a high linear energy transfer (LET) between 80–100 keV/µm. The short range is disadvantageous since particles need to be emitted directly at the position of the tumor cells to damage their DNA. Beta emitters have a lower LET of 0.2 keV/µm, but their higher range of up to 1 cm allows cross-fire to be emitted from neighboring cells [147,148]. Therefore, the main advantage of beta emitters such as iodine-131 over alpha-emitters such as astatine-211 is that, due to the long range and resulting cross-fire, beta particle emitting radiopharmaceuticals do not need to be present in every tumor cell to induce treatment-relevant damage [148]. However, investigations with iodine-131 labeled metaiodobenzylguanidine (MIBG) showed that smaller spheroids were less prone to therapy, suggesting that micrometastases might survive the treatment. The results of treatment in vivo corresponded with spheroid treatment [144,145]. To target smaller tumors, the radionuclide astatine-211 is suitable, as the range is much smaller but with higher LET. In combination with iodine-131, a range of sizes can be targeted [145]. Similar to that, the combination of the high-energy beta particle emitter yttrium-90 targeting larger sizes and the medium-energy beta particle emitter lutetium-177 targeting smaller sizes have proven to be complementary [149]. As a special type of radionuclide, copper-64 is as positron emitter applicable for diagnostic purposes and additionally emits high-LET Auger electrons, appropriate for targeted therapy approaches. The extremely short range of under 1 µm implies high damage to adjacent cell DNA but, at the same time, the necessity to enter every tumor cell [146,150].

To investigate effects of potential radiotherapeutics or other compounds on cell viability, spheroids can be re-incubated in media to allow for potential regrow. For simulating radiotherapy in clinical practice, the therapeutic agent can be administered several times [165]. Using spheroids in this stage of evaluation is suitable, as spheroids are easy to handle without extensive laboratory costs and they recapitulate clinical results, as described above. Furthermore, the possibility to administer the radiopharmaceutical several times while observing spheroid growth is favorable.

**Figure 4:**
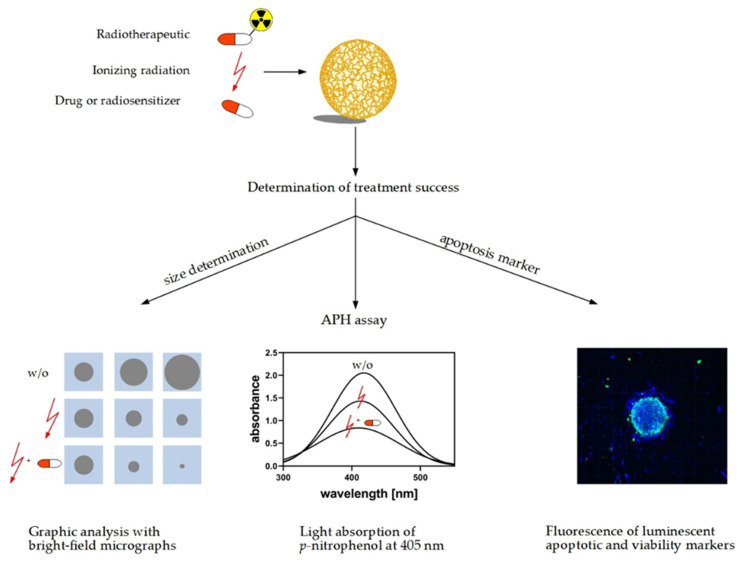
Methods for measuring response of spheroids to anticancer treatment: reactions of spheroids to treatments, e.g., irradiation, radiopharmaceuticals, or nonradioactive compounds, can be determined using these methods. The first option is to measure spheroid size using bright-field microscopy. Treatment effects are evaluated referring to shrinkage in size, reduction of growth rate, and capacity for regrowth (w/o: without). The second option is the acid phosphatase assay (APH) assay utilizing the activity of acid phosphatase in viable cells. The hydrolysis of *p*-nitrophenyl-phosphate to *p*-nitrophenol is detected as increase in absorption at 405 nm. The third option is to estimate the fraction of viable cells in a spheroid referring to staining of nucleic acids and viability markers such as DAPI and calcein, respectively.

Cell viability or rather treatment response of spheroids can be measured before and after drug testing, irradiation, or radiotherapy via different approaches (Figure 4). One of the easiest approaches that does not require dissociation of the spheroids is the acid phosphatase assay (APH). The assay is very sensitive and linear over a broad range of cell numbers (10^3^ to 10^5^ cells) [1,3,166,167]. Acid phosphatase hydrolyses *p*-nitrophenyl-phosphate to *p*-nitrophenol in viable cells. The absorption of *p*-nitrophenol can be measured at 405 nm and is proportional to its concentration [167]. The acid phosphatase assay is also suitable for quantification of viable cells during treatment testing, in particular, in spheroid cultures of pancreatic cancer cells [168]. Spheroids can be further examined taking into account the hallmarks of treatment response such as cell viability or integrity to be determined directly via immunostaining [140,169] or indirectly via monitoring growth and regrowth referring to spheroid size [136,163]. The size of the spheroid is measured through graphic analysis of bright field micrographs. Irradiated spheroids shrink in size followed by regrowth or disintegration depending on the applied dose. Short- and long-term effects can be evaluated referring to the relative decline or regrowth capacity of spheroids compared to nontreated controls. Application of radiosensitizing agents prior or during irradiation results in enhanced shrinkage and declined regrowth.

**Figure 5:**
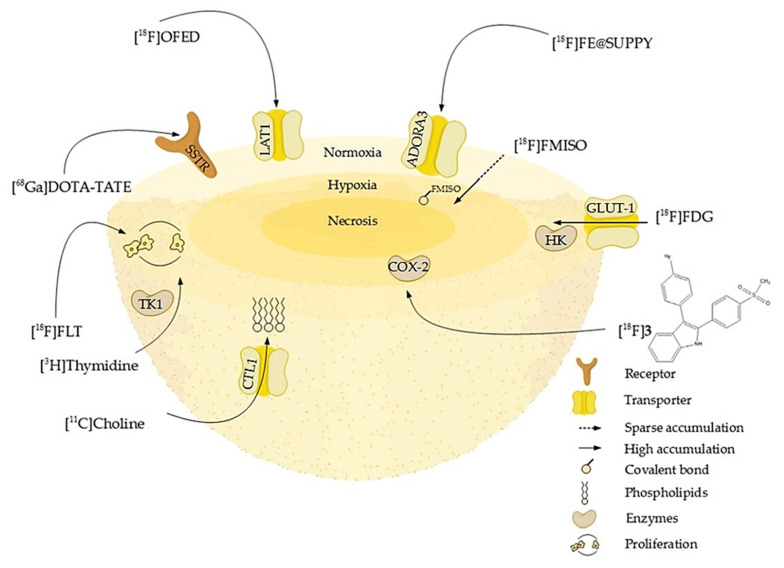
Schematic overview of molecular or pathophysiologic targets for well-established and novel radiotracers: some of them address membrane-bound cell receptors or transporters like somatostatin receptors (SSTR), adenosine receptors A_3_ (ADORA3), or L-amino-acid transporters (LAT1). Other tracers such as [^11^C]choline are taken up by transporters as the choline transporter-like protein (CTL1) and incorporated into the phospholipids of proliferating cells. The enzyme substrates [^3^H]thymidine and [^18^F]FLT target proliferating cells by incorporation into the DNA via for example the cytosolic thymidine kinase (TK-1). Enzyme inhibitors, portrayed here by [^18^F]3, a radiolabeled selective cyclooxygenase 2 inhibitor, in turn accumulate in areas of high enzyme concentration and activity. The radiolabeled glucose derivative [^18^F]FDG visualizes glucose turnover by glucose transport (GLUT-1) and hexokinase (HK) reaction and consequently the metabolic activity and viability of cells, which is high in the normoxic proliferating regions of the spheroid and lower in the hypoxic core. In contrast, [^18^F]FMISO binds to macromolecules in a hypoxic environment and thus accumulates in the hypoxic core (Table 4).

Prior to applications in animal models, the performance of newly developed radiotracers can be evaluated in 3D models. Vice versa, well-established radiotracers can be used to functionally characterize the pathophysiologic molecular and metabolic alterations in tumors using 3D models. For example, intrinsic tumor hypoxia is one of the major triggers for the induction of angiogenesis primarily via HIF-regulated genes including vascular endothelial growth factor (VEGF). This plays a central role in the switch from avascular to vascular growth in many tumors [170]. Hypoxia not only has been shown to activate several pathways via stabilization of the transcription factors associated with tumor progression HIF-1α and HIF-2α but also has been identified as a critical parameter of the tumor microenvironment. It affects therapeutic efficacy of various treatment modalities including radiotherapy and oxygen-dependent chemotherapeutic approaches. Monitoring hypoxia in individual human tumors to optimize treatment schemes, e.g., for intensity-modulated radiotherapy planning, is thus a new challenge for clinicians. To visualize hypoxic regions in tumors, a variety of PET tracers such as [^18^F]fluoromisonidazole ([^18^F]FMISO), [^18^F]fluoroazomycinarabinoside ([^18^F]FAZA), or [^64^Cu]copper(II)-diacetyl-bis(N(4)-methylthio-semicarbazone ([^64^Cu]ATSM) are currently under investigation [171,172,173,174]. At low oxygen concentrations, nitroimidazoles such as [^18^F]FMISO are intracellularly reduced by nitroreductases and trapped depending on the formation of covalent adducts of reduced nitroimidazoles to intracellular macromolecules [175]. [^18^F]FMISO uptake indicates the presence of inherent hypoxic areas with pO_2_ < 10 mmHg oxygen, which has been described to be the limit for the fixation of misonidazoles [176]. Other radiotracers do not visualize hypoxia directly but target specific enzymes or receptors overexpressed in tumors (Figure 5). The comparison between investigations on spheroids and in vivo provided essential background information. Therefore, Monazzam et al. [165] suggested to evaluate novel radiotracer candidates first in spheroid models before performing PET tracer imaging in animals in order to minimize animal numbers and costs.

From a radiopharmaceutical point of view, it might also be feasible to measure radiotracer uptake in spheroids using radioluminography, a method that is often performed after intravenous injection of a radiotracer into tumor-bearing animals in order to determine its distribution. Here, after resection, freezing, and sectioning of organs and tumors, samples are placed on a radioluminographic imaging plate sensitive to β- or γ-emission. This can also be done with spheroids. After incubation with the radiolabeled compound, cryopreserved spheroids are sectioned and then evaluated using radioluminography [179,180]. As a result, evaluating the loco-regional uptake and enrichment of novel radiotracers in 3D models is possible [161]. Alternatively, uptake of radiotracers by spheroids can also be measured using a radioactivity counter [165,181]. After incubating with the tracer, the spheroids are washed and the radioactive uptake is measured through the emitted radiation. However, this method does not provide information on regional differences in radiotracer uptake in different cellular layers of a spheroid.

A great variety of radiotracers has been tested by different research groups on multiple tumor entities. Some of them were also tested on spheroids (Table 4). The selection reported here includes compounds radiolabeled with positron emitters (^11^C, ^18^F, and ^68^Ga), most of which are clinically established radiotracers. Other given examples such as [^18^F]3 and [^18^F]OFED are still under evaluation [24,182]. Moreover, ^3^H- and ^14^C-labeled compounds are also mentioned as examples; however, their use is restricted to preclinical research. The selection of radiotracers depends on the tumor entity and expression of target receptors. An example is [^68^Ga]Ga-DOTATATE that can be used as somatostatin analogue for targeting somatostatin receptors (SSTR) 2 and 5 [183] in SSTR overexpressing neuroendocrine tumors (NETs) [184]. Besides other NETs, also pancreatic NET can be diagnosed with [^68^Ga]Ga-DOTATATE [185]. For radiopharmacists, the mini-panel PET scanner-based microfluidic radiobioassay system developed by Liu et al. [186] is of interest. Microfluidic radiobioassays are assays using radiotracers to detect samples on chips. Liu et al. [186] extended this approach with the use of PET to detect the radiopharmacokinetics in 3D models.

## 10. Conclusions and Perspective

In summary, 3D models exhibit important characteristics resembling tumors in vivo that are absent in monolayer cultures, in particular with respect to the genetic stability of the cells, their spatial organization, formation of gradients and barriers, better cell-cell and cell-matrix communication, and overall higher resistance to irradiation and chemotherapy. This is especially true for tumor stroma entities as heterogeneous as PDAC. Spheroids can be cultivated with the help of scaffold materials such as Matrigel™ or hyaluronic acid, or they can be cultivated without scaffolding materials in low-adhesion plates, hanging drops, or rotating vessels. More sophisticated approaches are 3D models on chips or strips that allow for direct measurements and co-culture spheroids that reflect the tumor microenvironment. Together with spheroids, organoids are of great interest in cancer research. They do not originate from cell lines but from resected tumor tissue and can be used for personalized medicine together with genetic profiling. Three-dimensional models not only can be used as in vitro models but also can be translated into in vivo models via subcutaneous or orthotopic transplantation into a suitable recipient organism, usually mice. This allows, amongst other things, the modeling of precursor lesions. For example, by orthotopic transplantation of PDAC-originating organoids, the formation of tumors from the PanIN lesion to the pancreatic adenocarcinoma has been demonstrated in detail. Especially for drug development, 3D models are now state-of-the-art. They allow both negative and positive selection of drug candidates, even in high-throughput scenarios, before animal experiments are performed. This reduces not only the number of laboratory animals but also the costs of drug development. Three-dimensional models can also be used to pursue new approaches in radiopharmaceutical and radiobiological cancer research. This allows for investigations on specific aspects important for establishing new radiotracers such as tissue perfusion, specific and nonspecific radiotracer uptake, and locoregional radiotracer enrichment. The influence of physicochemical and pathophysiological parameters, such as interstitial pressure, pH-value, and hypoxic and necrotic regions can also be modelled and investigated reliably. With regard to the aspect of chemo- and radioresistance, spheroids, in particular co-culture spheroids, resemble natural tumors more closely than monolayer cultures. Repeated experiments in the course of the growth of spheroids can be carried out and can allow for a more precise evaluation of radiooncological therapy approaches. However, the evaluation of novel diagnostic radiotracers and new approaches in radiotherapy has not yet been performed on PDAC 3D models and just rarely for radiopharmaceuticals. Our aim is to stimulate and strengthen an in-depth discussion of this exciting topic, especially from the point of view of radiopharmacists. In our view, tumors that prove to be refractory or resistant to classical therapies, especially conventional radiotherapy, are predestined for radiotheranostic applications. Requirements are suitable molecular targets, chemically available radiotheranostics that address the target with appropriate selectivity and affinity and the ability to combine them with chemotherapeutic or radiosensitizing agents. Radionuclide-based diagnostics initially provide information on the individual molecular signature of the respective tumor and metastases. Later on, they offer essential information for monitoring the molecular behavior of the tumor during therapy. In the case of heterogeneous tumor-stroma entities as PDAC, obtaining knowledge of the molecular signature of the tumor-associated cells and extracellular matrix as well as the addressability of the target structures is essential. Consequently, therapeutic concepts have to address the tumor-associated cells and extracellular matrix as well or even primarily. Ultimately, these concepts have to be studied in animal and clinical experiments; however, the 3D models discussed here offer an opportunity to test the feasibility of new radiotheranostic approaches in vitro. Very promising in this context are approaches for both high-throughput analysis and automation. Summarizing, the stronger inclusion of 3D models such as spheroids; organoids; or, in particular, co-culture systems in radiotracer and radiotherapeutic research should be taken into account, especially for PDAC-oriented approaches. Specifically addressing the tumor or stroma-associated molecular processes and remodeling could be the key to success in this tumor entity.

## Figures and Tables

**Table 1:** Overview of current techniques for 3D models used in pancreatic ductal adenocarcinoma (PDAC) research.

3D Model	Technique	Literature
**Multicellular spheroids**	Scaffold: Matrigel and collagen	[34,35,36,37]
	Scaffold: Methylcellulose	[38,39]
	NanoCulture plates	[40]
	Hyaluronan/chitosan coated plates	[41]
	Ultralow attachment plates	[42]
	concave polydimethylsiloxane microwell plates	[43]
	Hanging drop	[38,44,45]
**Organoids**	Mechanical and chemical dissociation	[46,47,48,49]
**Co-cultures**	Cultivation with stromal cells	[42,50]
	Cultivation with stellate cells	[51]
	Triple culture with fibroblasts and monocytes	[52,53]
	Triple culture with fibroblasts and endothelial cells	[54]
**Biotechnical microsystems**	Microfluidic chip	[55]
	Microfluidic plate	[56]
	HepaChip^®^	[57,58]
	Magnetic bioprinting	[59]

**Table 2:** Three-dimensional models for PDAC research and their application.

3D Model	Field of Interest	Literature
Multicellular spheroids	Protein levels	[40,65,66]
	Hypoxia marker	[38]
	Drug screening	[42,43,67,68]
	MicroRNA expression	[43]
	qRT-PCR	[43]
Organoids	KRAS or other genetic mutation	[46,69]
	Orthotopic transplantation	[48,70,71]
	Subcutaneous transplantation	[70]
	PanIN development	[48]
Co-cultures	Invasive behavior	[50,70]
	Therapy resistance	[68,72,73]
	Migration	[41,74]
	EMT marker expression	[74]
	Proliferation	[73]
	T-cell inhibition	[52]
	Protein expression	[73]
	Signaling pathways	[37]
Biotechnical microsystems	Drug response	[56,57,58]
	Marker and factor expression	[56]
	Hypoxia	[75]

**Table 3:** Examples for radiopharmaceuticals that have been used on 3D models of various tumor entities and partly in combination with radiosensitizers or external radiation.

Therapeutics	Tumor Entity	Target	Treatment Combinations	Literature
[^211^At]MABG	neuroblastoma	norepinephrine receptor		[151]
[^131^I]MIGB	glioma neuroblastoma	norepinephrine receptor	radiosensitizer Disulfiram	[145,152,153]
[^131^I]MIP-1145	melanoma	melanin uptake	radiosensitizer topotecan, AG014699	[154]
[^125^I]IUdR	glioblastoma	DNA (S-phase)		[138]
[^177^Lu]Lu-DOTATATE	pancreatic neuroendocrine tumor, lung cancer	somatostatin analogue	radiosensitizer onalespib	[155]
[^225^Ac]DTPA	glioblastoma	DNA		[156]
[^213^Bi]C595 AC	prostate cancer, pancreatic cancer	mucin1		[157,158,159]
[^213^Bi]BLCA-38 AC	prostate cancer	unknown glycoprotein		[157]
[^213^Bi]PAI2 AC	prostate cancer	urokinase plasminogen activator		[157]
[^213^Bi]7.16.4	breast cancer	HER-2/*neu*		[160]
[^213^Bi]Mab 13A	murine breast cancer	CD44		[161]
[^90^Y]Mab 13A	murine breast cancer	CD44		[161]
[^90^Y]cetuximab	human squamous cell carcinoma models	epidermal growth factor receptor	External radiation	[162]
[^90^Y]C225	Head and neck squamous cell carcinoma	epidermal growth factor receptor	External radiation	[163]
[^212^Pb]mAb 376.96	pancreatic ductal adenocarcinoma	B7-H3 (CD276)		[164]

Isotope half-life: [^90^Y]: 2.7 days, [^131^I]: 8 days, [^125^I]: 59.5 days, [^177^Lu]: 6.7 days, [^212^Pb]: 10.64 h, [^213^Bi]: 45.6 min, [^211^At]: 7.2 h, and [^225^Ac]: 10 days. [^211^At]MABG: meta-[^211^At]astatobenzylguanidine, [^131^I]MIGB: Meta-[^131^I]iodobenzylguanidine, [^131^I]MIP-1145: *N*-(2-diethylaminoethyl)-4-(4-fluoro-benzamido)-5-iodo-2-methoxy-benzamide, [^125^I]IUdR: ^125^I-deoxyuridine, [^90^Y]C225: [^90^Y]Y-CHX-A′′-DTPA-C225, [^177^Lu]LuDOTATATE: [^177^Lu]Lu-(Tyr3)octreotate, and [^225^Ac]DTPA: [^225^Ac]-diethylenetriaminepentaacetic acid, AG014699: Rucaparib.

**Table 4:** Selected examples for established radiotracers and radiotracers still in experimental research that have been used on 3D models of various tumor entities.

Tracer	Tumor Entity	Application	Reference
[^18^F]FDG	pheochromocytoma, breast cancer, colorectal adenocarcinoma, colorectal carcinoma, glioblastoma, colon carcinoma	Glucose turnover, viability, metabolic activity	[24,165,172,177]
[^18^F]FMISO	colorectal adenocarcinoma, colorectal carcinoma, melanoma	Hypoxia	[172,173,174]
[^18^F]OFED	pheochromocytoma	Large neutral amino-acid transporter	[24]
[^18^F]FE@SUPPY	colorectal adenocarcinoma, colorectal carcinoma	A_3_-Adenosine receptor	[172]
[^18^F]3	colorectal adenocarcinoma	Cyclooxygenase-2	[173]
[^18^F]FLT	breast cancer, glioblastoma, colon carcinoma	Proliferation	[165]
[^18^F]FAZA	colon adenocarcinoma, lung squamous cell carcinoma, lung adenocarcinoma	Hypoxia	[171]
[^68^Ga]Ga-DOTATATE	pheochromocytoma	Somatostatin receptor 2/5	[24]
[^3^H]Methionine	rectal adenocarcinoma	Protein synthesis	[178]
[^3^H]Thymidine	rectal adenocarcinoma	Proliferation	[178]
[^14^C]FDG	rectal adenocarcinoma	Glucose turnover, viability, metabolic activity	[178]
[^11^C]Methionine	breast cancer, glioblastoma, colon carcinoma	Protein synthesis	[165]
[^11^C]Choline	breast cancer, glioblastoma, colon carcinoma	Membrane lipid synthesis	[165]
[^64^Cu]ATSM	colon adenocarcinoma, lung squamous cell carcinoma, lung adenocarcinoma	Hypoxia	[171]

Isotope half-life: [^3^H]: 12.3 years, [^14^C]: 5730 years, [^11^C]: 20.3 min, [^18^F]: 109.8 min, [^64^Cu]: 12.7 h, and [^68^Ga]: 67.4 min. Abbreviation: [^18^F]FDG: 2-[^18^F]fluoro-2-deoxyglucose; [^18^F]FMISO: [^18^F]fluoromisonidazole; [^18^F]OFED: *O*-3-(2[^18^F]fluoroethoxy)-4-hydroxyphenylalanine; [^18^F]FE@SUPPY: 5-(2-[^18^F]fluoroethyl)2,4-diethyl-3-(ethylsulfanyl-carbonyl)-6-phenylpyridine-5-carboxylate; [^18^F]3: 3-(4-[^18^F]fluorophenyl)-2-(4-methylsulfonylphenyl)-1H-indole; [^18^F]FLT: 3′-deoxy-3′-[^18^F]fluorothymidine; [^18^F]FAZA: 1-(5-fluoro-5-deoxy-α-D-arabinofuranosyl)-2-nitroimidazole; [^68^Ga]GaDOTA-TATE: [^68^Ga]-1,4,7,10-tetraazacyclododecane-*N,N′,N″,N‴*-tetraaceticacid-D-Phe^1^,Tyr^3^-octreotate; and [^64^Cu]ATSM: [64Cu]copper-diacetyl-bis[*N*(4)-methylthiosemicarbazone].
